# Vital Signs: Changes in Opioid Prescribing in the United States, 2006–2015

**DOI:** 10.15585/mmwr.mm6626a4

**Published:** 2017-07-07

**Authors:** Gery P. Guy, Kun Zhang, Michele K. Bohm, Jan Losby, Brian Lewis, Randall Young, Louise B. Murphy, Deborah Dowell

**Affiliations:** ^1^Division of Unintentional Injury Prevention, National Center for Injury Prevention and Control, CDC; ^2^Division of Toxicology and Human Health Sciences, Agency for Toxic Substances and Disease Registry, CDC; ^3^Division of Population Health, National Center for Chronic Disease Prevention and Health Promotion, CDC.

## Abstract

**Background:**

Prescription opioid–related overdose deaths increased sharply during 1999–2010 in the United States in parallel with increased opioid prescribing. CDC assessed changes in national-level and county-level opioid prescribing during 2006–2015.

**Methods:**

CDC analyzed retail prescription data from QuintilesIMS to assess opioid prescribing in the United States from 2006 to 2015, including rates, amounts, dosages, and durations prescribed. CDC examined county-level prescribing patterns in 2010 and 2015.

**Results:**

The amount of opioids prescribed in the United States peaked at 782 morphine milligram equivalents (MME) per capita in 2010 and then decreased to 640 MME per capita in 2015. Despite significant decreases, the amount of opioids prescribed in 2015 remained approximately three times as high as in 1999 and varied substantially across the country. County-level factors associated with higher amounts of prescribed opioids include a larger percentage of non-Hispanic whites; a higher prevalence of diabetes and arthritis; micropolitan status (i.e., town/city; nonmetro); and higher unemployment and Medicaid enrollment.

**Conclusions and Implications for Public Health Practice:**

Despite reductions in opioid prescribing in some parts of the country, the amount of opioids prescribed remains high relative to 1999 levels and varies substantially at the county-level. Given associations between opioid prescribing, opioid use disorder, and overdose rates, health care providers should carefully weigh the benefits and risks when prescribing opioids outside of end-of-life care, follow evidence-based guidelines, such as *CDC’s Guideline for Prescribing Opioids for Chronic Pain,* and consider nonopioid therapy for chronic pain treatment. State and local jurisdictions can use these findings combined with Prescription Drug Monitoring Program data to identify areas with prescribing patterns that place patients at risk for opioid use disorder and overdose and to target interventions with prescribers based on opioid prescribing guidelines.

## Introduction

In 2015, drug overdoses accounted for 52,404 deaths in the United States, 63.1% of which involved an opioid (*1*). Among opioid-related deaths, approximately 15,000 (approximately half) involved a prescription opioid (*2*). In addition, an estimated 2.0 million persons in the United States had opioid use disorder (addiction) associated with prescription opioids in 2015 (*3*). The economic burden of prescription opioid overdose, abuse, and dependence is estimated to be $78.5 billion each year in the United States (*4*). Prescription opioid-related overdose deaths and admissions for treatment of opioid use disorder have increased in parallel with increases in opioids prescribed in the United States, which quadrupled from 1999 to 2010 (*5*). This increase was primarily because of an increase in the use of opioids to treat chronic noncancer pain (*6*,*7*). Previously, opioids had primarily been reserved for severe acute pain, postsurgical pain, and end-of-life care. This change in prescribing practice increased the amount of opioids prescribed for three reasons. First, opioid use for chronic noncancer pain increased the number of opioid prescriptions. Second, the use of opioids to treat ongoing chronic conditions increased the average lengths of time for which opioids were prescribed (*6*,*7*). Third, average dosages of opioid prescriptions tend to be higher for patients who are prescribed opioids for long periods of time, effectively increasing the average amount of opioids supplied per prescription (*6*,*7*). Together, these changes placed more persons at risk for opioid use disorder and overdose (*8*–*11*).

Chronic pain is one of the most common reasons for seeking medical attention in the United States, and prescription opioids are frequently prescribed to manage pain (*12*). However, opioids should only be used when benefits are expected to outweigh risks. Ensuring that patients have access to safe, effective treatment is critical and involves improving the way opioids are prescribed. To improve understanding of opioid prescribing trends in the United States before the release of *CDC’s 2016 Guideline for Prescribing Opioids for Chronic Pain* (Guideline), CDC analyzed changes in national and county-level opioid prescribing and characteristics associated with higher prescribing rates at the county-level (*13*).

## Methods

Data on opioid prescribing come from the QuintilesIMS Transactional Data Warehouse, which provides estimates of the number of opioid prescriptions dispensed in the United States based on a sample of approximately 59,000 pharmacies, representing 88% of prescriptions in the United States.

Changes in opioid prescribing at the national level were analyzed from 2006 to 2015. Prescribing rates included overall opioid prescribing rates, high-dose prescribing rates, and prescribing rates by days’ supply (≥30 days and <30 days). Annual opioid prescribing rates were calculated by dividing the number of opioid prescriptions by the U.S. Census population estimates each year. High-dose prescribing rates include prescriptions with daily dosage ≥90 morphine milligram equivalents (MME) (*13*). All rates are per 100 persons. Additional measures included MME per capita, average daily MME per prescription, and average days’ supply per prescription. Cold and cough products containing opioids and buprenorphine products indicated for conditions other than pain were excluded.

To determine where prescribing changes occurred, opioid prescribing at the county level was examined in 2010 (when prescribing levelled off nationally) and 2015. Quartiles were created using MME per capita to characterize the distribution of opioids prescribed. The percentage of counties experiencing changes in opioid prescribing measures from 2010 to 2015 was calculated. A change of ≥10% was considered to be an increase or decrease, whereas changes <10% were considered stable. County-level characteristics were examined in 2015 by MME per capita quartiles. County characteristics were obtained from the U.S. Census Bureau (age, urban/rural status); American Community Survey (race/ethnicity, percent uninsured, percent unemployed, income); U.S. Diabetes Surveillance System (diabetes prevalence); Dartmouth Atlas of Health Care (provider supply); Centers for Medicare and Medicaid Services (Medicaid and Medicare coverage); Behavioral Risk Factor Surveillance System (arthritis prevalence); and the Area Health Resource File (percent disabled, suicide rate). To identify county-level factors associated with MME per capita in 2015, a stepwise multivariable linear regression model incorporating age, race/ethnicity, insurance status, education, unemployment rates, poverty rates, median income, urban/rural status (metropolitan, micropolitan [i.e., town/city; nonmetro], and noncore [i.e., rural; nonmetro]), suicide rates, dentist and primary care physician density, and diabetes, arthritis, and disability prevalence was estimated.

## Results

**FIGURE 1:**
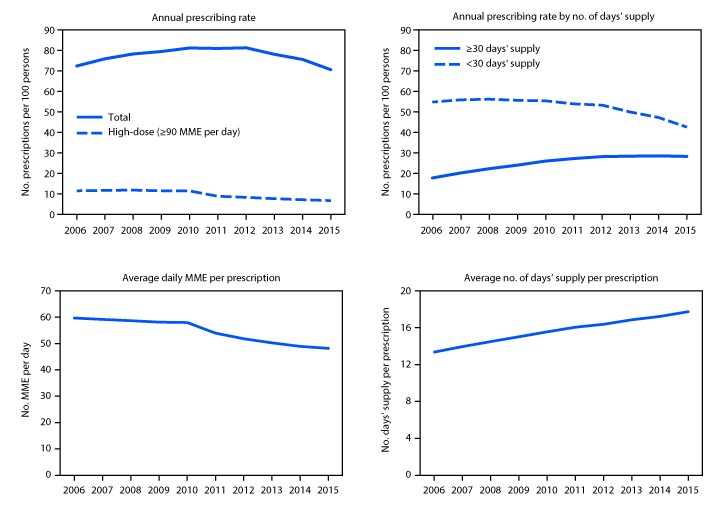
Annual opioid prescribing rates, by number of days’ supply, average daily morphine milligram equivalent (MME) per prescription, and average number of days’ supply per prescription — United States, 2006–2015

In the United States, annual opioid prescribing rates increased from 72.4 to 81.2 prescriptions per 100 persons from 2006 to 2010, were constant from 2010 to 2012, and then decreased by 13.1% to 70.6 per 100 persons from 2012 to 2015 (Figure 1). Annual high-dose opioid prescribing rates remained stable from 2006 to 2010 and then declined by 41.4% from 11.4 per 100 persons in 2010 to 6.7 in 2015. Annual prescribing rates for prescriptions of ≥30 days’ supply increased 58.9% from 17.6 per 100 persons in 2006 to 28.0 per 100 persons in 2012 and leveled off from 2012 to 2015. Annual prescribing rates for prescriptions of <30 days’ supply were stable from 2006 to 2012 and decreased 20.2% from 53.2 per 100 persons in 2012 to 42.4 in 2015. Average daily MME per prescription remained stable from 2006 to 2010 and then decreased 16.9% from 58.0 in 2010 to 48.1 in 2015. Average days’ supply prescribed increased 33.0% from 13.3 in 2006 to 17.7 in 2015.

**FIGURE 2:**
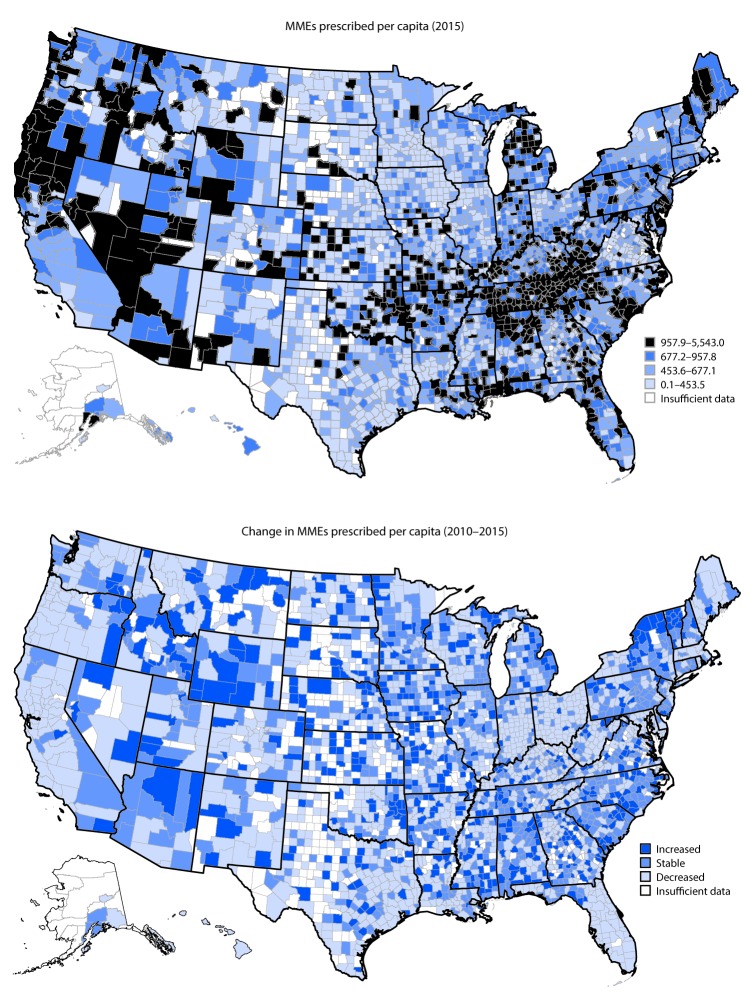
Morphine milligram equivalents (MMEs) of opioids prescribed per capita in 2015 and change in MMEs per capita during 2010–2015, by county — United States, 2010–2015

From 2010 to 2015, the amount of opioids prescribed in the United States decreased from 782 to 640 MME per capita (data not shown). In 2010 and 2015, the amount of opioids prescribed across counties varied substantially (Figure 2). From 2010 to 2015, among counties with sufficient data MME per capita decreased in 49.6% of counties, remained stable in 27.8% of counties, and increased in 22.6% of counties (Table 1). Overall prescribing rates decreased in nearly half (46.5%) of counties, whereas high-dose opioid prescribing rates and average daily MME per prescription decreased in the majority of counties, with 86.5% and 72.1% of counties, respectively, experiencing decreases. From 2010 to 2015, average number of days’ supply increased in 73.5% of counties.

**TABLE 1:** Percentage of counties with changes* in opioid prescribing — United States, 2010–2015

Opioid prescribing measures	Decrease (%)	Stable (%)	Increase (%)
MME per capita	49.6	27.8	22.6
Overall prescribing rate	46.5	33.8	19.6
High-dose^†^ prescribing rate	86.5	6.7	6.9
Average daily MME per prescription	72.1	25.7	2.2
Average days’ supply per prescription	1.1	25.4	73.5

**Abbreviation:** MME = morphine milligram equivalent.

* Among counties with sufficient data, changes of ≥10% were considered to represent an increase or decrease, whereas changes of <10% were considered stable.

^†^ High-dose prescribing rates include prescriptions with daily dosage ≥90 MME.

Despite reductions in prescribing, the amount of opioids prescribed in 2015 remained high relative to 1999 levels and varied substantially across the country, from an average of 203 MME per capita in the lowest quartile to 1,319 MME per capita in the highest quartile. Opioid prescribing amounts varied across several county-level characteristics (Table 2). After adjustment in the multivariable model, the following characteristics were associated with higher amounts of opioids prescribed: a larger percentage of non-Hispanic whites; higher rates of uninsured and Medicaid enrollment, lower educational attainment; higher rates of unemployment; micropolitan status; more dentists and physicians per capita; a higher prevalence of diagnosed diabetes, arthritis, and disability; and higher suicide rates. Together, these factors explain approximately 32% of the variation in the amount of opioids prescribed at the county-level.

**TABLE 2:** Sociodemographic characteristics of counties by MME per capita quartiles* — United States, 2015

Characteristics	Total	Lowest Quartile	Second Quartile	Third Quartile	Highest Quartile	Adjusted Results^†^	
						Coefficient	p-value
**Population no. (%)**	**—**	**76,225,923 (23.8)**	**108,825,101 (33.9)**	**83,254,830 (26.0)**	**52,330,662 (16.3)**	**—**	**—**
**Average MME per capita**	**—**	**202.9**	**528.5**	**776.9**	**1,318.7**	**—**	**—**
**Age group, yrs (%)**							
<35	43.3	43.2	44.6	43.3	42.1	NA	—
35–64	38.8	38.6	38.7	38.9	39.0	NA	—
≥65	17.9	18.2	16.7	17.7	18.9	NA	—
**Race/Ethnicity (%)**							
Non-Hispanic white	80.1	76.9	78.3	81.8	83.6	6.9	<0.001
Non-Hispanic black	9.0	9.3	9.4	9.3	8.0	NA	—
Hispanic^§^	7.0	9.5	8.3	5.3	4.8	NA	—
Other	3.9	4.4	4.0	3.7	3.6	NA	—
**Insurance status (%)**							
Uninsured	14.9	15.3	14.3	14.5	15.7	7.5	<0.001
Medicare	16.8	17.2	15.8	16.7	17.7	NA	—
Medicaid	20.6	19.2	19.3	20.7	23.3	5.3	<0.001
**Education level (%)**							
No high school diploma	16.9	17.3	15.9	16.1	18.4	6.9	<0.001
**Employment level (%)**							
Unemployed	7.6	6.7	7.3	7.9	8.5	11.0	<0.001
**Income**							
Income below the Federal Poverty Level (%)	15.5	15.3	14.5	15.2	17.1	−3.8	0.08
Median annual income ($)	22,479	22,339	23,747	22,612	21,216	NA	—
**Urban/Rural (%)^¶^**							
Metropolitan	38.5	29.5	47.9	41.9	34.7	0.6	0.003
Micropolitan	21.6	13.6	20.2	24.9	27.6	1.3	<0.001
Noncore	39.9	56.9	31.9	33.2	37.7	NA	—
**Provider density per 100,000 residents**							
Primary care physicians (no.)	55.2	44.1	57.4	59.5	60.0	2.1	<0.001
Dentists (no.)	38.2	30.5	41.5	41.3	39.5	4.0	<0.001
**Disease/Condition prevalence (%)**							
Diagnosed diabetes	11.1	10.2	10.6	11.4	12.1	30.5	<0.001
Diagnosed arthritis	24.8	23.7	23.9	25.4	26.3	9.6	0.009
Disabled	15.1	14.4	13.5	15.3	17.4	21.9	<0.001
**Selected death rate**							
Suicides per 100,000 (no.)	11.3	7.7	15.1	13.5	9.0	10.4	<0.001

**Source**: U.S. Census Bureau (age, urban/rural status), American Community Survey (race/ethnicity, percent uninsured, percent unemployed, income), U.S. Diabetes Surveillance System (diabetes prevalence), Dartmouth Atlas of Health Care (provider supply), Centers for Medicare and Medicaid Services (Medicaid and Medicare coverage), Behavioral Risk Factor Surveillance System (arthritis prevalence), and the Area Health Resource File (percent disabled, suicide rate).

**Abbreviations:** MME = morphine milligram equivalents; NA = not applicable (variable was not included in the final model).

* Quartiles were created using MME per capita to characterize the distribution of opioids prescribed.

^†^ Results are from a stepwise multivariable linear regression model of the continuous variable, county-level MME per capita.

^§^ Hispanic persons could be of any race.

^¶^ The three classification levels for counties were 1) metropolitan: part of a metropolitan statistical area 2) micropolitan: part of a micropolitan statistical area (has an urban cluster of ≥10,000 but <50,000 population); and 3) noncore: not part of a metropolitan or micropolitan statistical area.

## Discussion

The amount of opioids prescribed in the United States began to decrease in 2011. However, in 2015, at 640 MME per capita, it remains approximately three times as high as in 1999, when 180 MME per capita were sold in the United States (*5*), and nearly four times as high as the amount distributed in Europe in 2015 (*14*).

Two prescribing changes appear to be associated with the decrease in MME prescribed per capita in the United States from 2010 to 2015. First, average daily MME per prescription decreased after 2010, both nationwide and in most counties. The largest decreases occurred from 2010 to 2012, following publication of two national guidelines defining high-dose opioid prescribing as >200 MME/day (*15*,*16*). It also coincided with studies demonstrating progressively increasing overdose risk at prescribed opioid dosages exceeding 20, 50, and 100 MME per day (*9*–*11*) and publications highlighting associations of prescribed opioids with overdose deaths (*5*,*17*). Second, the rate of opioid prescribing decreased nationwide and in many counties. Nationally, opioid prescribing rates leveled off from 2010 to 2012, and then decreased by 13.1% from 2012 to 2015. These decreases might reflect growing awareness among clinicians and patients of the risks associated with opioids. Throughout this period, however, the average duration of opioid prescriptions increased, in part because of the continued increase in longer opioid prescriptions (≥30 days) through 2012, followed by a stabilization of the rate, and a substantial decrease in shorter prescriptions (<30 days) after 2012. This pattern, along with the trends in overall numbers of opioid prescriptions, might reflect fewer patients initiated on opioid therapy after 2012, whereas patients already receiving opioids were more likely to continue receiving them. Patients are at risk for continuing opioids long-term once they have received them for >5 days (*18*), and are unlikely to discontinue opioids after they have received them for 90 days (*19*), highlighting both the importance of minimizing unnecessary initial opioid exposure and potential challenges in reducing opioid use among patients already receiving them.

From 2010 to 2015, half of counties in the United States experienced reductions in the amount of opioids prescribed, with substantial decreases in certain states. In 2011 and 2012, Ohio and Kentucky, respectively, mandated that clinicians review Prescription Drug Monitoring Program (PDMP) data and implemented pain clinic regulation (*20*). MME per capita decreased in 85% of Ohio counties and 62% of Kentucky counties from 2010 to 2015. In Florida, where multiple interventions targeted excessive opioid prescribing from 2010 through 2012, (e.g., pain clinic regulation and mandated PDMP reporting of dispensed prescriptions) (*21*), the amount of opioids prescribed per capita decreased in 80% of counties from 2010 to 2015. During this time, Florida also experienced reductions in prescription opioid-related overdose deaths (*21*).

Despite reductions, the amount of opioids prescribed in 2015 remained high relative to 1999 levels and varied substantially across the country, with average per capita amounts prescribed in the top quartile of counties approximately six times the amounts prescribed in the lowest quartile. Larger amounts were prescribed in micropolitan counties and in counties with a higher prevalence of diagnosed diabetes and arthritis. The latter finding might represent treatment for pain associated with these or co-occurring painful conditions. However, there are effective nonopioid treatments for pain whose benefits outweigh the harms (*13*). Reasons for higher opioid use in micropolitan counties might include less access to quality health care and other treatments for pain, such as physical therapy. In addition, persons in rural areas might travel to micropolitan areas, which often serve as an anchor community for a much larger rural region, to receive medical care and pick up medications.

Despite reductions in opioid prescribing in recent years, opioid-involved overdose death rates continue to increase. However, these increases have been driven largely by use of illicit fentanyl and heroin (*1*). There is no evidence that policies designed to reduce inappropriate opioid prescribing are leading to these increases. Combined implementation of mandated provider review of PDMP data and pain clinic laws reduced the amount of opioids prescribed, prescription opioid-involved overdose deaths, and all opioid-involved deaths (*20*). The policies were also associated with reductions in heroin overdose deaths that were not statistically significant (*20*). By reducing the number of persons exposed to opioids and the subsequent risk of opioid use disorder these policies might reduce the number of persons initiating illicit opioid use in the longer term (*20*).

The findings in this report are subject to at least four limitations. First, QuintilesIMS estimates of dispensed prescriptions have not been validated, and they do not include prescriptions dispensed directly by prescribers (although this likely represents a small minority of prescribed opioids), potentially biasing opioid prescribing downwards. Second, county-level analyses are aggregated by the county where an opioid is dispensed, and cannot account for prescriptions obtained by persons outside of the county. Third, the analysis does not include clinical outcomes. However, previous analyses have found associations between population-level amounts of opioids prescribed and opioid overdose death rates (*5*), and between prescribed dosages and individual overdose risk (*9*–*11*). Finally, because data on the indications for which opioids were prescribed were not available, the appropriateness of opioid prescriptions, or whether opioids were prescribed for acute, chronic, or end-of-life pain, could not be determined.

Although some variation in opioid prescribing is associated with characteristics such as the prevalence of possibly painful conditions (e.g., arthritis), differences in these characteristics explain only a fraction of the wide variation in opioid prescribing across the United States. This variation suggests inconsistent practice patterns and a lack of consensus about appropriate opioid use and demonstrates the need for better application of guidance and standards around opioid prescribing practices (*13*). CDC’s Guideline provides evidence-based recommendations about opioid prescribing for primary care clinicians treating adult patients with chronic pain outside of active cancer treatment, palliative care, and end-of-life care (*13*). The Guideline can help providers and patients weigh the benefits and risks for opioids according to best available evidence and individual patients’ needs and safely taper opioids if risks outweigh benefits. The Guideline recommends the use of nonopioid therapies, such as acetaminophen, nonsteroidal anti-inflammatory medications, exercise therapy, and cognitive behavioral therapy for chronic pain (*13*).

Given associations between opioid prescribing, opioid use disorder, and opioid overdose rates (*5*), states and local jurisdictions can use these findings to target high-prescribing areas for interventions such as academic detailing for clinicians or individual educational visits to clinicians (*22*), and increased access to medication-assisted treatment for patients with opioid use disorder. Innovative approaches such as virtual physical therapy sessions with pain coping skills training (*23*,*24*) can be used to improve access to effective treatment for chronic pain. In addition, states can consider policies that can reduce opioid overdose, including mandated PDMP use and pain clinic laws (*20*). Changes in opioid prescribing can save lives. The findings of this report demonstrate that substantial changes are possible and that more are needed.

**Key Points**

• The amount of opioids prescribed in the United States peaked in 2010 and then decreased each year through 2015. Despite reductions, the amount of opioids prescribed remains approximately three times as high as in 1999.

• Opioid prescribing varied substantially across the country, with average per capita amounts prescribed in the top-prescribing counties approximately six times the amounts prescribed in the lowest prescribing counties in 2015.

• Higher amounts of opioids were prescribed in counties with a larger percentage of non-Hispanic whites; a higher prevalence of diabetes and arthritis; micropolitan counties; and counties with higher rates of unemployment and Medicaid enrollment.

• The substantial variation in opioid prescribing observed at the county-level suggests inconsistent practice patterns and a lack of consensus about appropriate opioid use and demonstrates the need for better application of guidance and standards around opioid prescribing practices.

• Health care providers can follow the *CDC’s Guideline for Prescribing Opioids for Chronic Pain*, which provides evidence-based recommendations about opioid prescribing for primary care clinicians treating adult patients with chronic pain, outside of active cancer treatment, palliative care, and end-of-life care.

• Additional information is available at https://www.cdc.gov/vitalsigns/.
